# Sensitive detection methods are key to identify secondary *EGFR* c.2369C>T p.(Thr790Met) in non-small cell lung cancer tissue samples

**DOI:** 10.1186/s12885-020-06831-3

**Published:** 2020-05-01

**Authors:** Cleo Keppens, Elisabeth M. C. Dequeker, Etienne Rouleau, Nils ’t Hart, Lukas Bubendorf, Kelly Dufraing, Céline Garrec, Paul Guéguen, Aude Lamy, Antonio Marchetti, Patrick Pauwels, Ales Ryska, Véronique Tack, Luigi Tornillo, Kaat Van Casteren, Jan H. von der Thüsen, Karen Zwaenepoel, Birgit Lissenberg-Witte, Erik Thunnissen, Ed Schuuring

**Affiliations:** 1 Department of Public Health and Primary Care, Biomedical Quality Assurance Research Unit, University of Leuven, Leuven, Belgium; 2 Service de Génétique des Tumeurs, Gustave Roussy, Villejuif Cedex, France; 3 Department of Pathology, University of Groningen, University Medical Center Groningen (UMCG), Hanzeplein 1, PO Box 30001, 9700 RB Groningen, the Netherlands; 4 Department of Pathology, Isala, Zwolle, The Netherlands; 5 Institute of Pathology, University Hospital Basel, Basel, Switzerland; 6 Institut de Biologie, CHU Hôtel Dieu, Laboratoire de Génétique Moléculaire, Nantes Cedex 1, France; 7 CHRU Brest/Hôpital Morvan, Laboratoire de Génétique Moléculaire et d’Histocompatibilité, Brest, France; 8 CHU de Rouen / Hôpital Charles Nicolle, laboratoire de génétique somatique des tumeurs, Rouen Cedex, France; 9 Laboratory of Molecular Diagnostics, Center for Advanced Studies and Technology, University of Chieti, 66100 Chieti, Italy; 10 Department of Pathology, University Hospital Antwerp, Edegem, Belgium; 11 Centre for Oncological Research (CORE), University of Antwerp, Edegem, Belgium; 12 Department of Pathology, Charles University Medical Faculty Hospital, Hradec Kralove, Czech Republic; 13 GILAB, Allschwil, AG Switzerland; 14 Department of pathology, Erasmus Medical Center Rotterdam, Rotterdam, The Netherlands; 15 Amsterdam UMC, Vrije Universiteit Amsterdam, Department of Epidemiology and Biostatistics, Amsterdam, The Netherlands; 16 Department of pathology, VU University Medical Center (VUMC) Amsterdam, Amsterdam, the Netherlands

**Keywords:** Non-small cell lung cancer, External quality assessment, Predictive biomarker, *EGFR*, c.2369C>T p.(Thr790Met), Resistance, Osimertinib

## Abstract

**Background:**

Correct identification of the *EGFR* c.2369C>T p.(Thr790Met) variant is key to decide on a targeted therapeutic strategy for patients with acquired EGFR TKI resistance in non-small cell lung cancer. The aim of this study was to evaluate the correct detection of this variant in 12 tumor tissue specimens tested by 324 laboratories participating in External Quality Assessment (EQA) schemes.

**Methods:**

Data from EQA schemes were evaluated between 2013 and 2018 from cell lines (6) and resections (6) containing the *EGFR* c.2369C>T p.(Thr790Met) mutation. Adequate performance was defined as the percentage of tests for which an outcome was available and correct. Additional data on the used test method were collected from the participants. Chi-squared tests on contingency tables and a biserial rank correlation were applied by IBM SPSS Statistics version 25 (IBM, Armonk, NY, USA).

**Results:**

In 26 of the 1190 tests (2.2%) a technical failure occurred. For the remaining 1164 results, 1008 (86.6%) were correct, 151 (12.9%) were false-negative and 5 (0.4%) included incorrect mutations. Correct p.(Thr790Met) detection improved over time and for repeated scheme participations. In-house non-next-generation sequencing (NGS) techniques performed worse (81.1%, *n* = 293) compared to non-NGS commercial kits (85.2%, *n* = 656) and NGS (97.0%, *n* = 239). Over time there was an increase in the users of NGS. Resection specimens performed worse (82.6%, *n* = 610 tests) compared to cell line material (90.9%, *n* = 578 tests), except for NGS (96.3%, *n* = 344 for resections and 98.6%, *n* = 312 for cell lines). Samples with multiple mutations were more difficult compared to samples with the single p.(Thr790Met) variant. A change of the test method was shown beneficial to reduce errors but introduced additional analysis failures.

**Conclusions:**

A significant number of laboratories that offer p.(Thr790Met) testing did not detect this relevant mutation compared to the other EQA participants. However, correct identification of this variant is improving over time and was higher for NGS users. Revising the methodology might be useful to resolve errors, especially for resection specimens with low frequency or multiple variants. EQA providers should include challenging resections in the scheme.

## Background

The mutational status of the epidermal-growth factor receptor (*EGFR*) gene is used as a predictive biomarker for treatment with targeted therapy in patients with advanced non-small cell lung cancer (NSCLC) [1–5]. Demonstrating the presence of an activating *EGFR* mutation is of crucial importance in considering the use of EGFR-tyrosine kinase inhibitors (TKIs). These TKIs have shown improved progression-free survival of patients with *EGFR*-mutated NSCLC [1–3], which lead to their approval by the European Medicines Agency or Food and Drug Administration [6, 7]. The inhibitors include reversible first-generation (e.g., gefitinib and erlotinib) and irreversible second-generation drugs (e.g., afatinib and dacomitinib).

Despite high initial response rates to first and second generation EGFR-TKIs, eventually all patients with advanced NSCLC harbouring an *EGFR* mutation will progress on these treatments due to acquired resistance, with a median progression-free survival of 9.7–13.1 months [8, 9]. The most common mechanism of acquired resistance to first and second generation EGFR-TKIs is the *EGFR* c.2369C>T p.(Thr790Met) variant (often referred to as ‘T790M’), ranging from 51 to 68% [10–13]. Mechanistically, this base substitution leads to replacement of a threonine by a methionine resulting in steric hindrance for binding of these TKIs to the tyrosine kinase domain, and decreased TKI effectivity.

Correct, reproducible and timely identification of the c.2369C>T p.(Thr790Met) variant is important at the time of relapse for appropriate treatment selection. Patients with the specific *EGFR* c.2369C>T p.(Thr790Met) variant are eligible for treatment with osimertinib, which also irreversibly targets this variant [14, 15].

In tumor specimens from patients with relapse of NSCLC after EGFR-TKI treatment, the fraction of mutant alleles with the c.2369C>T p.(Thr790Met) mutation is nearly always lower than that of the initial *EGFR* mutation, and an appropriate and sufficiently sensitive method for *EGFR* analysis is necessary [16]. A variety of technologies have been reported to have a range of sensitivities between 62 and 100% [17, 18]. The Therascreen EGFR RGQ PCR Kit (Qiagen), Cobas EGFR Mutation Test v2 (Roche) and FoundationOne CDx (Foundation Medicine) have been approved by the FDA for the detection of *EGFR* mutations as companion diagnostics for tissue specimens [19]. In addition, numerous CE-IVD tests are becoming available for predictive testing including the relevant *EGFR* mutations.

Laboratories are required to participate in external quality assessment (EQA) ring studies to regularly demonstrate their performance of predictive testing, and to compare their test methods according to well-documented validation or verification procedures [20]. Several European EQA programs have reported on the performance of predicitive testing for both activating and resistant clinically relevant *EGFR* mutations in tumor tissue samples for individual laboratories and different technologies [21–27].

The aim of this study is to present a longitudinal overview of the performance to correctly detect the c.2369C>T p.(Thr790Met) variant with regard to the test method, sample type and variant allele frequency (VAF). For this purpose, we collected results from all samples with *EGFR* c.2369C>T p.(Thr790Met) variants from yearly EQA programs for *EGFR* analysis in NSCLC between 2013 and 2018, organized by the European Society of Pathology (ESP) and the French national Gen&Tiss consortium [28, 29].

## Methods

Formalin-fixed paraffin embedded (FFPE) material was distributed for further DNA extraction. For the ESP schemes, tissue slides of both resection specimens and established cell lines were sent with a thickness of minimum 4–5 μm. Tissue blocks with fixed and embedded cell lines distributed in 2013 and 2014 were created in-house (**Supplemental Table** **1**), while the cell line material in 2017 was purchased from Horizon Discovery (Cambridge, UK). In the other years, resection specimens from leftover patient material was collected and samples were sectioned at a central preparation laboratory. For the Gen&Tiss schemes only cell lines were provided as 1 to 3 FFPE curls (extraction of minimum 400 ng DNA) ordered from Horizon. For all samples, the presence and VAF of the c.2369C>T p.(Thr790Met) variant were validated prior to distribution with next-generation sequencing (NGS) by an ISO15189-accredited reference laboratory [20] with experience in molecular pathology. A detailed overview of the distributed samples to in total 324 participants between 2013 and 2018 is represented in Table 1. All data used in this manuscript were retrieved from the *EGFR* EQA scheme for NSCLC, between 2013 and 2018 (ESP) and in 2014 and 2016 (Gen&Tiss).

**Table 1:** Overview of the samples distributed during the EQA schemes between 2013 and 2018

Provider	Scheme year	Sample type	1^st^***EGFR*** Variant	VAF variant 1 (in %)	2^nd^ (and 3^rd^) ***EGFR*** variant	VAF variant 2 (in %)	# participants	# correct (%)	# false-negative (%)	# wrong mutation (%)	# technical failure (%)
ESP	2013	Cell line	c.2369C>T p.(Thr790Met)	25^b^	c.2573T>G p.(Leu858Arg)	25^b^	107	65 (70.7)	27 (29.3)	0 (0.0)	15 (14.0)
	2014	Cell line	c.2369C>T p.(Thr790Met)	45^b^	c.2573T>G p.(Leu858Arg)	45^b^	144	133 (92.4)	10 (6.9)	1 (0.7)	0 (0.0)
		Cell line		25^b^	c.2573T>G p.(Leu858Arg)	25^b^	144	135 (93.8)	8 (5.6)	1 (0.7)	0 (0.0)
		Resection		27	c.2155G>A p.(Gly719Ser) + c.2327G>A p.(Arg776His)	79 76	144	77 (53.5)	66 (45.8)	1 (0.7)	0 (0.0)
	2015	Resection	c.2369C>T p.(Thr790Met)	15	c.2573T>G p.(Leu858Arg)	89	114	92 (84.4)	16 (14.7)	1 (0.9)	5 (4.4)
	2016	Resection	c.2369C>T p.(Thr790Met)	19	c.2235_2249del p.(Glu746_Ala750del)	30	43	42 (97.7)	1 (2.3)	0 (0.0)	0 (0.0)
	2017 (Jun.)^a^	Resection	c.2369C>T p.(Thr790Met)	18	c.2573T>G p.(Leu858Arg) + c.2389T>A p.(Cys797Ser)	35 18	107	87 (81.3)	20 (18.7)	0 (0.0)	0 (0.0)
	2017 (Oct.)^a^	Cell line	c.2369C>T p.(Thr790Met)	20	/	/	102	97 (99.0)	1 (1.0)	0 (0.0)	4 (3.9)
	2018	Resection	c.2369C>T p.(Thr790Met)	22	c.2573T>G p.(Leu858Arg)	22	101	101 (100.0)	0 (0.0)	0 (0.0)	0 (0.0)
		Resection		43	c.2236_2248delinsCAAC p.(E746_A750delinsQP)	80	101	101 (100.0)	0 (0.0)	0 (0.0)	0 (0.0)
Gen&Tiss	2014	Cell line	c.2369C>T p.(Thr790Met)	17	/	/	43	39 (92.8)	2 (4.8)	1 (2.4)	1 (2.3)
	2016	Cell line	c.2369C>T p.(Thr790Met)	22	c.2573T>G p.(Leu858Arg)	18	40	39 (100.0)	0 (0.0)	0 (0.0)	1 (2.5)

ESP schemes: all samples were provided on glass slides, Gen&Tiss schemes: cell lines were provided as curls from a cytoblock. For each sample, the percentage of correct, false-negative results, wrong mutations and technical failures is based on c.2369C>T p.(Thr790Met) detection irrespective of the performance to detect the additional variants. Technical failures are represented with respect to the total number of tests. Correct results, false-negatives and wrong mutations are calculated in relation to the total number of analyzable tests (total tests minus technical failures). Refseq *EGFR*: LRG_304t1 (NM_005228.5). ^a^The 2017 ESP scheme was organized in 2 distribution rounds (one in June and one in October). ^b^Variant allele frequency based on the percentage of tumor cells. E.g. cell line of 50% tumor cells in a wild-type background was considered as a VAF of 25%. Abbreviations: *EGFR* Epidermal growth factor receptor; *ESP* European Society of Pathology; *LRG* Locus Reference Genomic; *VAF* variant allele frequency; #, number; /, No second variant and VAF given as sample only contains c.2369C>T p.(Thr790Met)

Participants were required to analyze these samples using their routine procedures within 14 calendar days and to provide information on the applied methodologies along with their testing results in an online datasheet. Results were assessed by a team of international experts in molecular pathology using pre-defined and harmonized scoring criteria [21, 22].

For this study, datasheet entries of EQA cases with the c.2369C>T p.(Thr790Met) variant were classified into four categories: (i) technical failures for which no outcome could be reported because of a test failure, (ii) correct identification of the c.2369C>T p.(Thr790Met) variant, (iii) false-negative results, i.e. failure to detect the c.2369C>T p.(Thr790Met) variant in the sample, (iv) wrong mutation, in case the mutation was detected but was incorrectly reported e.g. c.2155G>T p.(Thr790Met) instead of c.2369C>T p.(Thr790Met). Technical failures are represented with respect to the total number of tests. Correct results, false-negatives and wrong mutations were calculated in relation to the total number of analyzable tests (total tests minus technical failures). Adequate performance was defined as the percentage of tests for which an outcome was available and correct (second category). The *EGFR* reference sequence applied throughout this manuscript for description of the variants is Locus Regerence Genomic (LRG) LRG_304t1 (NM_005228.5).

In total, three samples were excluded from the analyses: two samples were below the pre-defined VAF cut-off of 10% (one case with VAF of 1% and one with VAF 6%, respectively), and one sample was excluded because it was not tested by all participants, yielding a total of 1190 tests.

Statistics were performed using IBM SPSS Statistics version 25 (IBM, Armonk, NY, USA). Differences in the percentage of correct entries, false-negatives, wrong mutations or technical failures were calculated by contingency tables (Chi-squared tests or Fisher’s exact tests for cell counts below 5) for a given category. Significance levels were set at α = 0.05. For correlation of the VAF with c.2369C>T p.(Thr790Met) outcome and technical failures, a ranked biserial correlation was performed with the occurrence of an error (false-negative or wrong mutation) or technical failure as dichotomous variable (present versus not present) and VAF as ordinal variable.

## Results

From 2013 to 2018, 12 samples contained c.2369C>T p.(Thr790Met), on which in total 1190 tests were performed (Table 1). For 26 (2.2%) tests no outcome was reported because of a technical failure. These 26 failures were reported by 25 individual laboratories in mostly cell line cases, with the majority of reasons being that the DNA concentration was too low for analysis (**Supplemental Table** **2**). For the remaining 1164 analyses, 1008 (86.6%) were adequate (outcome available and correct), 151 tests (13.0%) were false-negatives, and for 5 of 1164 results (0.4%) an incorrect mutation was reported by three different laboratories. Incorrect mutations consisted of c.2155G>T p.(Thr790Met) (three times), c.2174C>T p.(Thr725Met) (once) and c.2369C>T p.(Tyr790Met) (once).

For the 12 different cases distributed during the different scheme years, the lowest performances were observed for one cell line in 2013 (70.7%, *n* = 107) and one resection specimen in 2014 (53.5%, *n* = 144) (Table 1).

**Fig. 1:**
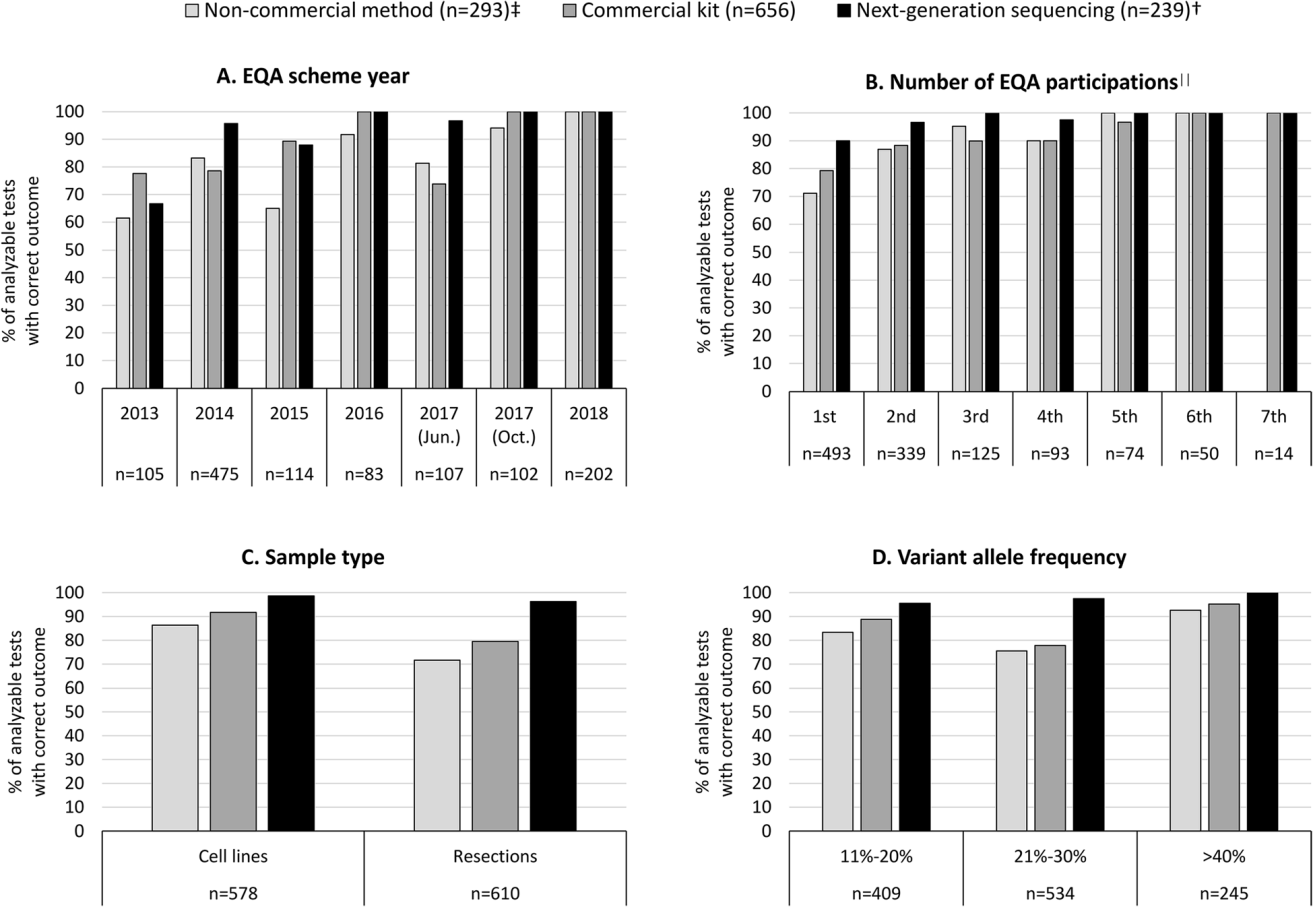
Percentage of analyzable tests with correct c.2369C>T p.(Thr790Met) identification for the different technique types. 2/1190 tests were excluded as no method information was available, bringing the total analyzed tests on 1188. The two excluded tests were performed in 2013 during a 1^st^ EQA participation, on cell line material with an allele frequency of 25%, and resulted in one correct result and one technical failure. Correct results are calculated in relation to the total number of analyzable tests (total tests minus technical failures). †The category ‘next-generation sequencing’ includes both commercial and in-house panels. ‡Non-commercial methods include in-house sequencing methods that are non-NGS based. ∥The first and second distribution round of the Lung 2017 scheme are counted as two separate participations (4 months apart). The detailed percentage of correct results, false-negatives, wrong mutations and technical failures is given in **Supplemental Table****3**. Abbreviations: EQA, external quality assessment; NGS, next-generation sequencing

Over time, an overall improvement in correct c.2369C>T p.(Thr790Met) detection was observed, from 70.3% (*n* = 105 tests) in 2013 to 100.0% (*n* = 202 tests) in 2018 (Fig. 1**, panel a**). The three different technique types also improved over time, being commercial kits (*n* = 656), NGS (both commercial and non-commercial panels) (*n* = 239), and non-commercial non-NGS sequencing methods such as dideoxy or Sanger sequencing (*n* = 293) (Fig. 1**, panel a**).

In addition, the ability to detect the c.2369C>T p.(Thr790Met) variant was compared to the number of participations in EQA schemes. In total, 493 tests were carried out during the laboratories’ first time participation in EQA, and 77.9% of these tests resulted in a correct detection of c.2369C>T p.(Thr790Met). Laboratories who participated in the EQA scheme for the sixth or seventh time in a row performed 50 and 14 predictive tests respectively, and in all of these tests (100.0%) the c.2369C>T p.(Thr790Met) variant was correctly identified (Fig. 1**, panel b**).

Resection specimens performed significantly less (82.6%, *n* = 610 tests) compared to cell line material (90.9%, *n* = 578 tests). This was especially the case for non-commercial methods (86.3%, *n* = 189 for cell lines versus 71.6%, *n* = 104 for resections) and commercial kits (79.5%, *n* = 344 for resections versus 91.7%, *n* = 312 for cellines), but not for NGS (96.3%, *n* = 162 for resections and 98.6%, *n* = 77 for cell lines) (Fig. 1**, panel c**). Samples with a VAF between 21 and 30%, performed overall significantly worse, while samples with a VAF of more than 40% performed significantly better. For NGS users, the VAF did not seem to affect the performance as much as compared to the other technique types (Fig. 1**, panel d**). A further biserial rank correlation revealed that false-negative results and wrong mutations were correlated to lower VAFs (rrb = 0.058, *p* = 0.048) in cases for which no technical failures occurred (*n* = 1163) (**Supplemental Figure****1****, panel A**). Considering all tests (*n* = 1188), no correlation was observed between the occurrence of technical failures and the VAF (rrb = − 0.053, *p* = 0.068) (**Supplemental Figure****1****, panel B**). The detailed percentages of correct results, false-negatives, wrong mutations and technical failures per technique type for the different EQA scheme years, number of participations, sample types and VAFs is given in **Supplemental Table** **3**.

**Fig. 2:**
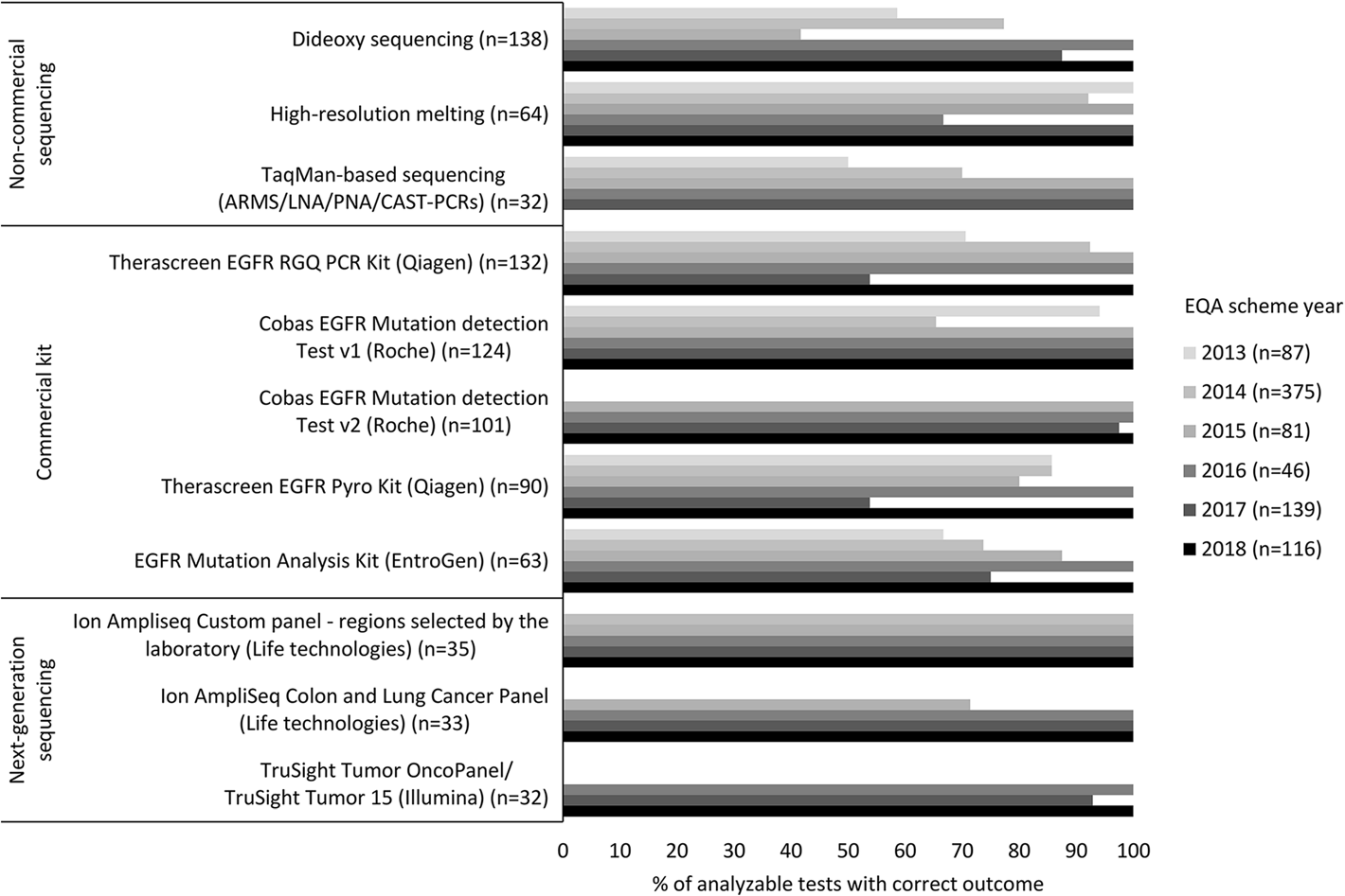
Percentage of analyzable tests with correct outcome over time for the 11 most frequently used c.2369C>T p.(Thr790Met) detection methods. Correct results are calculated in relation to the total number of analyzable tests (total tests minus technical failures). Analysis methods are represented as reported by the participants in the electronic datasheets. The category ‘next-generation sequencing’ includes both commercial and in-house panels. Non-commercial methods include in-house sequencing methods that are non-NGS based. The detailed percentage of correct results, false-negatives, wrong mutations and technical failures for these 11 methods is given in **Supplemental Table** **4**. Abbreviations: ARMS, Amplification Refractory Mutation System; CAST, Competitive allele-specific TaqMan; *EGFR*, epidermal growth factor receptor; LNA, locked nucleic acid; PCR, polymerase chain reaction; PNA, peptide nucleic acid

In Fig. 2**,** the performance of scheme years 2013–2018 were summarized for the most frequently used methodologies. The results of all commercial test kits improved over time. Both Therascreen (EGFR RGQ and pyro) kits displayed a lower performance in 2017 (both 53.8%) not observed for the other methods, which was resolved in 2018. In 2014, performance of the Cobas v1 kit was the lowest, but increased to 100.0% correct identification in 2015, along with the introduction of the v2 kit with a similarly high performance. Both commercial and non-commercial NGS methods were introduced between 2014 and 2016 and displayed very good performances during the first years of use. The most prevalent non-commercial non-NGS tests also showed an improvement, but were less frequently used in the most recent scheme years (Fig. 2). The detailed percentages of of correct results, false-negatives, wrong mutations and technical failures for the 11 most widely used techniques depicted in Fig. 2 are given in **Supplemental Table** **4**.

The overall performance of the various methods is shown in **Supplemental Table** **5**. A wide variety of test methodologies was used for every technique type, with varying performances ranging from 46.2 to 100.0%. Between 2013 (*n* = 105) and 2018 (*n* = 202), an increase of samples tested by NGS was observed from 4.7 to 38.6%, at the expense of a decrease of non-commercial sequencing methods from 41.9 to 9.9%. The percentage of commercial kits remained stable with 53.3 and 51.5% in the first and last scheme year, respectively. Note that the performance of non-commercial in-house sequencing techniques was significantly lower (81.1%, *n* = 293 tests, *p* < 0.05) compared to commercial kits (85.2%, *n* = 656 tests) and NGS (97.0%, *n* = 239 tests).

**Fig. 3:**
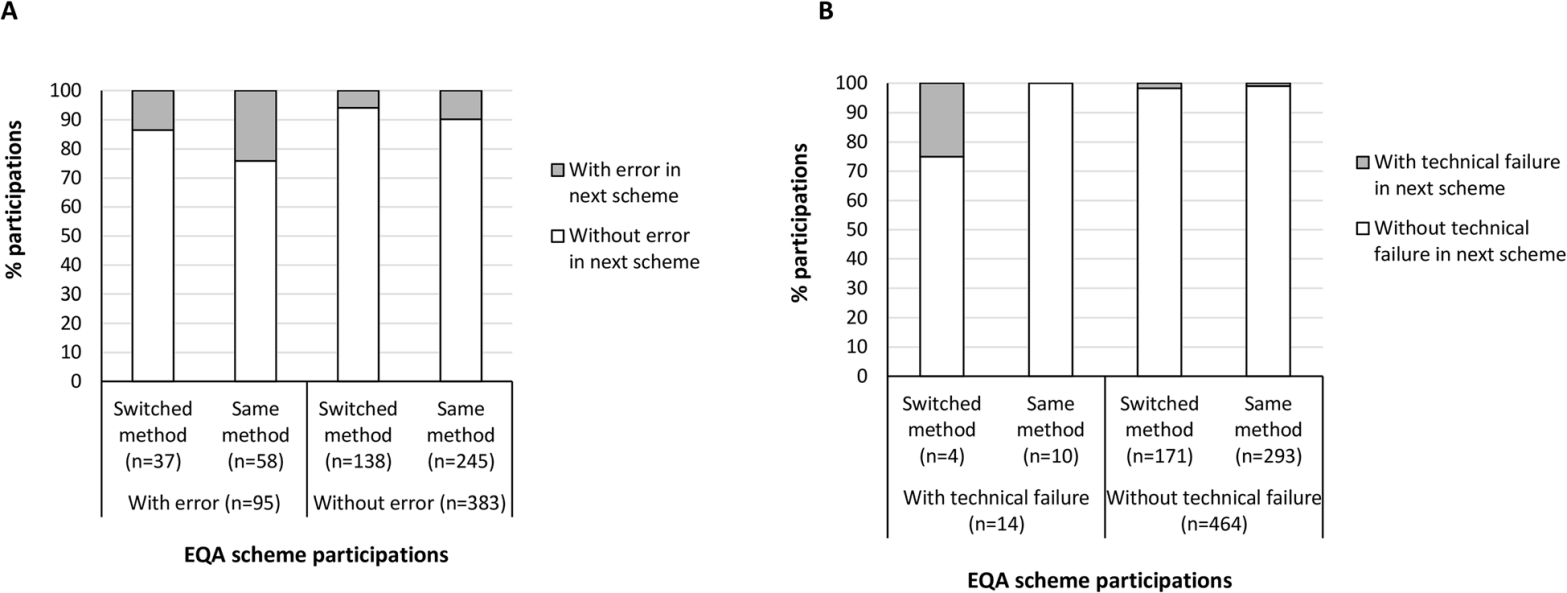
Influence of a change in test method on incorrect results (panel **a**) and technical failures (panel **b**). Eight hundred one individual participations were considered of which 323 participations were excluded because they were first participations for which no method information from the previous participation was available. Incorrect results include participations during which a false-negative or wrong mutation was reported. The evaluation of the effect of a switch in test method was evaluated on laboratory level for a given participation. Switching to a new methodology included both a switch of technique type (e.g. from a commercial test kit to NGS) as an upgrade to a higher version number for a given methodology, given that this might affect the detection limit and variants included in the test. Abbreviations: EQA, external quality assessment. Chi-squared tests or Fisher’s Exact tests (for cell counts below 5) were used to assess significance

To evaluate the influence of a change in test methodology within laboratories, we evaluated 478 performed tests. In 303 of the 478 tests (63.4%), the participants used the same test method as during their previous participation. Participants who did not change their methods and made an error in the EQA scheme, more frequently reported a false-negative or wrong mutation in the next scheme (Fig. 3**, panel a**). In contrast, participants who reported a technical failure and switched methods between two EQA schemes, more often were inable to resolve their technical failure during the next scheme (Fig. 3**, Panel b**).

## Discussion

We performed a longitudinal analysis of the different testing methods used by European EQA participants to detect the c.2369C>T p.(Thr790Met) variant in tissue samples, and demonstrated a wide variety of methodologies and percentages of false-negative results.

A lower performance was observed for non-commercial sequencing techniques compared to commercial test kits and NGS. This is not surprising given that the sensitivity to detect *EGFR* mutations in tissue has been reported to be on average 20% for in-house sequencing techniques (such as Sanger sequencing) compared to 1–5% for commercial kits and NGS [17, 18]. Results from other EQA schemes also revealed higher error rates for c.2369C>T p.(Thr790Met) compared to other mutations [21, 22] depending on the used methodology. In the national Italian EQA scheme, performance was lower for direct sequencing compared to other techniques, and follow-up confirmed our results that the switch to more sensitive methods could increase the percentage of good performers [23, 24]. The individual studies reported above evaluated the performance to detect multiple *EGFR* variants at a certain time-point, with varying sample numbers and types [17]. Inadequate method information has often been reported as a study limitation [25]. This stresses the importance of this study with longitudinal data on *EGFR* c.2369C>T p.(Thr790Met) variant detection. We did not compare the differences between both EQA providers as only two cell line cases were included in the study for Gen&Tiss, and additional data is needed to make solid conclusions in this scheme.

For the Cobas EGFR v1 mutation detection kit, more false-negative results occurred in the 2014 EQA scheme (**Supplemental Table** **4**). The majority of errors in 2014 occurred for the resection specimen which contained two additional variants besides c.2369C>T p.(Thr790Met), stressing the importance of including such samples in EQA schemes. The high number of false-negatives were communicated to the manufacturer, after which the kit was adapted and no false-negative results occurred in the next scheme.

In addition, commercial kits in 2017 produced more false-negative results, caused by the lower performance of the Therascreen RGQ and Pyro kits (both 46.2% false-negative results reported on 13 performed tests). However, the false-negative rate dropped to 0% in 2018. This was also the case for all other methods in 2018, and the properties of the distributed samples cannot be neglected as an influencing factor. Moreover, several other methods, such as the Cobas EGFR v2 (for plasma and tissue testing) and NGS-based methods were predominantly used in later scheme years, and influences of improved technology and knowledge over time cannot be discarded.

A switch to more sensitive and high-troughput techniques has been previously advised [2]. Current recommendations state that laboratories testing for c.2369C>T p.(Thr790Met) should deploy assays capable of detecting clinically relevant mutations in as little as 5% of *EGFR* alleles, based on the analytical sensitivity of allele specific real-time polymerase chain reaction in the clinical trials for third generation TKIs [2]. This was reflected by our study with an apparent increase over time from non-commercial testing methods to more sensitive methods such as NGS.

In NSCLC, the clinical relevance of the *EGFR* c.2369C>T p.(Thr790Met) variant at a VAF of 1% in a tumor-rich tissue sample is still under debate, and ultimate therapy decisions by the treating physician need to consider the complete clinical patient context. For this study, the threshold of variants to be detected was set at 10%, given that this VAF should at least be detected by the majority of method detection thresholds in tissue, allowing the comparison with the earlier scheme years. The possibility of small subclones in routine practice (even as little as 5%), and the upcoming use of circulating tumor DNA when insufficient tissue or tumor cells are present, stresses the importance of low VAF detection. This is exemplified for the two excluded samples below the 10% cut-off with false-negative rates of 22.2% (*n* = 54, VAF = 6%) and 56.1% (*n* = 41, VAF = 1%).

In this study, the switch of a laboratory to another testing method (irrespective of the specific technique or assay type) had a positive effect on the false-negative or wrong mutation rates (Fig. 3). These findings emphasize the educational value of EQA participation and the individual feedback that is provided afterwards. However, switching of test method resulted in more technical failures. This suggests that when a new method is adopted, laboratories are still in a learning phase concerning optimal analysis conditions, and both aspects (errors versus technical failures) require a different approach. This is in contrast with previously reported findings, in which changing the test method for KRAS proto-oncogene GTPase (*KRAS)* analysis did not influence the EQA score [27]. One explanation is that the detection of *KRAS* mutations is restricted to few, well-defined mutations and single nucleotide polymorfisms in contrast to more complex *EGFR* mutations.

One sample with two additional *EGFR* variants (c.2155G>A p.(Gly719Ser) and c.2327G>A p.(Arg776His)) displayed the lowest performance, together with a case with an additional variant c.2573T>G p.(Leu858Arg). For the latter, the lower performance could be explained by the fact that this sample was distributed in the first year (2013) of this EQA scheme and many laboratories were still experiencing technical failures. This is supported by the fact that this case contributes to 15 of 26 reported failures, and that other samples with the same variants displayed a better performance in later schemes. For the first case, the presence of more than one additional mutation besides c.2369C>T p.(Thr790Met) requires extra caution, given that primer sequences need to be chosen specifically enough to prevent binding to nearby regions. This is especially the case for c.2369C>T p.(Thr790Met), as this variant has been reported to occur in less than 5% of treatment naïve primary lung cancers, but in 50% of treated NSCLC as a concomitant resistance variant in combination with another sensitizing *EGFR* variant [15].

Surprisingly, the performance for samples with a VAF in the lowest category of 11–20% was higher than that observed at a VAF of 21–30%. This could be explained by the fact that the category of 21–30% contained the two most difficult samples mentioned above with a performance score of 53.5% (case with 3 combined *EGFR* variants) and 70.7% (only case in the first scheme of 2013). Exclusion of these cases shifts the score to 87.3% (*n* = 409) for cases with a VAF of 11–20% and to 93.7% (*n* = 285) for cases with a VAF of 21–30%. An additional correlation indeed confirmed that lower VAFs were significantly related to more incorrect detection of c.2369C>T p.(Thr790Met) (**Supplemental Figure** **1****, panel a**), but not to the number of technical failures (**Supplemetal** Figure **1****, panel b**). However, the number of technical failures was low (25/1188), and these data need to be interpreted with caution.

For all categories, cell lines performed better compared to resection specimens, especially for non-commercial non-NGS methods. In contrast, NGS seems to perform excellently in both sample types (**Supplemental Table** **3**). Cell lines are included by EQA providers to track performance over time in a repeatable manner, to perform a technical scheme, or when resection material is scarce. Our findings now advocate the use of resection specimens, as cell lines seem to over-estimate the laboratory’s EQA performance, which could potentially provide a false sense of security. In addition, the ISO15189 [20] standard advizes to select an ISO17043 accredited scheme [29] that distributes material reflecting routine practice as close as possible.

The fact that resection specimens performed worse compared to cell lines and for users of non-commercial methods compared to commercial methods, could be explained by tissue heterogeneity, as different technologies require a different minimal number of neoplastic cells. Alternatively, the preanalytical factor of delay in transportation to the pathology department, or in resection specimen fixation may play a role [30], but is unlikely to occur with preparation of EQA cell lines.

One limitation of this study might be the omission of selection and estimation of the neoplastic cell percentage, which have been reported to be highly variable [31], and influence the quantity and quality of extracted DNA for further analysis. When evaluating the reasons for technical failures (**Supplemental Table** **2**), the majority of laboratories mention a too low percentage of neoplastic cells or DNA concentration. However, given the overall low frequency of technical failures, and the noticeable difference between methodologies for the provided cell lines (albeit less as compared to resection specimens), the analytical method seems to be of great importance, and EQA scheme participants are advised to re-check the suitability of their methods if deemed necessary.

While the percentage of false-negative results was 26% in 2013, no false-negative results were observed in 2018. This improvement might be attributed to the large increase in NGS users over time, which displayed an overall higher performance. Therefore, we also evaluated the percentage of correctly identified samples in relation to the number of successive EQA participations, irrespective of the used methodology. Results suggested that repeated EQA participation leads to improved performance, without the co-occurrence of a higher percentage of NGS users (data not shown). Nevertheless, molecular pathology is a permanently evolving field, and other factors should be considered, such as more experience with the distributed EQA material, or increased scientific knowledge of the involved staff by trainings and continuous education.

Despite the high response rates to various first and second generation EGFR-TKIs, eventually all patients with advanced NSCLC carrying an *EGFR* mutation, will progress due to acquired resistance [8, 9], most often due to the *EGFR* p.(Thr790Met) mutation [10–13]. Osimertinib was introduced as a third-generation EGFR-TKI that selectively and irreversibly targets the *EGFR* p.(Thr790Met) mutation. Currently, osimertinib is approved for treatment of p.(Thr790Met)-positive patients who have progressed on first or second generation EGFR-TKIs [14, 15]. However, in 2018 osimertinib was also approved as first-line therapy for advanced *EGFR*-mutated NSCLC regardless of p.(Thr790Met) mutation status [32]. In contract to the other first and second generation EGFR-TKIs, resistance mechanisms reported for osimertinib are very different and represent only 6–10% secondary *EGFR* mutations not including the p.(Thr790Met) mutation [33]. Although the p.(Thr790Met) mutation is not a major resistance mechanism for patients treated with osimertinib, our findings suggest that also for the detection of other double *EGFR* mutations, approved commercial panels and more sensitive detection methods should be used.

## Conclusions

Our findings illustrate an improvement over time in the detection of the *EGFR* c.2369C>T p.(Thr790Met) mutation mostly in combination with a second activating *EGFR* mutation in the same sample, related to an increased use of NGS. With the lower performance of non-commercial sequencing methods, it is advised to switch to approved commercial panels or to use more sensitive detection methods for testing of patient resections. A reconsideration of the test method and repeated participation has shown to be favorable in EQA, especially in resolving already existing errors. Participation in EQA programs could thus guide laboratories in identifying method-based shortcomings and in taking the necessary actions for improvement. EQA providers are advised to include challenging resection samples with a low VAF or multiple variants besides cell lines.

## Supplementary information

**Additional file 1 Supplemental Table S1**: Preparation of the in-house cell lines distributed in the 2013 and 2014 ESP EQA schemes

**Additional file 2 Supplemental Table S2**: Overview of applied test methods and reasons reported for the technical failures observed in the EQA schemes.

**Additional file 3 Supplemental Table S3**: Performance of different technique types related to time, number of EQA participations, sample type and variant allele frequency.

**Additional file 4 Supplemental Table S4**: Performance over time for the 11 most frequently used c.2369C > T p.(Thr790Met) detection methods.

**Additional file 5 Supplemental Table S5**: Use and performance of the different methods for c.2369C > T p.(Thr790Met) detection between 2013 and 2018.

**Additional file 6 Supplemental Figure S1**: Ranked biserial correlation between variant allele frequencies and incorrect outcomes (panel A) or technical failures (panel B).

## Data Availability

The datasets used and/or analysed during the current study are available from the corresponding author on reasonable request.

## Abbreviations

*EGFR*: Epidermal growth factor receptor
EQA: External quality assessment
ESP: European society of pathology
FFPE: Formalin-fixed paraffin embedded
NGS: Next-generation sequencing
LRG: Locus reference genomic
NM: Protein-coding transcripts (mRNA)
NSCLC: Non-small cell lung cancer
TKI: Tyrosine-kinase inhibitor
VAF: Variant allele frequency
