# Supplementary material for: Sensitive detection methods are key to identify secondary EGFR c.2369C>T p.(Thr790Met) in non-small cell lung cancer tissue samples
Source: BMC Cancer. 2020 May 1;20:366. doi: 10.1186/s12885-020-06831-3 (PMC7193365; doi:10.1186/s12885-020-06831-3)
Supplement: Supplementary file 1 — Additional file 1 Supplemental Table S1: Preparation of the in-house cell lines distributed in the 2013 and 2014 ESP EQA schemes [file 12885_2020_6831_MOESM1_ESM.pdf]

Supplemental Table 1: Preparation of the in-house cell lines distributed in the 2013 and 2014 ESP EQA schemes

Title	Sensitive detection methods are key to identify secondary EGFR c.2369C>T p.(Thr790Met) in non-small cell lung cancer tissue samples.
Journal	BMC Cancer
Authors	Cleo Keppens, Elisabeth MC Dequeker, Etienne Rouleau, Nils ‘t Hart, Lukas Bubendorf, Kelly Dufraing, Céline Garrec, Paul Guéguen, Aude Lamy, Antonio Marchetti, Patrick Pauwels, Ales Ryska, Véronique Tack, Luigi Tornillo, Kaat Van Casteren, Jan H von der Thüsen, Karen Zwaenepoel, Birgit Lissenberg-Witte, Erik Thunnissen, Ed Schuuring
Correspondence	Prof. dr. Ed Schuuring<br>Department of Pathology (HPC EA10)<br>University Medical Center Groningen<br>PO Box 30001, 9700 RB<br>Groningen, the Netherlands<br>Tel: (+31) 50 361 9623<br>Email: e.schuuring@umcg.nl

Supplemental table 1: Preparation of the in-house cell lines distributed in the 2013 and 2014 ESP EQA schemes

Provider	Sample type	Scheme year	Ratio *EGFR* mutated cell-line/*EGFR* wild-type cell line	1st *EGFR* Variant	VAF variant 1 (in %)	2nd *EGFR* variant	VAF variant 2 (in %)
ESP	Cell line	2013	50%/50%	c.2369C>T p.(Thr790Met)	25†	c.2573T>G p.(Leu858Arg)	25†
		2014	90%/10%	c.2369C>T p.(Thr790Met)	45†	c.2573T>G p.(Leu858Arg)	45†
			50%/50%		25†	c.2573T>G p.(Leu858Arg)	25†

In-house cell lines were created by mixing cell lines with the *EGFR* mutation with an *EGFR*-wild-type cell line in a ratio indicated in the respective column. The homogeneous mixed cells were fixed for one hour in neutral-buffered formalin, mixed with warm agar (all cells distributed in 4 tubes) and the agar plugs were embedded in paraffin-blocks conform standard histopathology procedures. Paraffin blocks were cut to sections with a thickness of 4-5 µm, and were provided on glass slides. Refseq EGFR: LRG_304t1 (NM_005228.4). †Variant allele frequency based on the percentage of tumor cells. E.g. cell line of 50% tumor cells in a wild-type background was considered as a VAF of 25%. Abbreviations: EGFR, epidermal growth factor receptor; ESP, European Society of Pathology; LRG, Locus Reference Genomic; VAF, variant allele frequency.
