# Supplementary material for: Sensitive detection methods are key to identify secondary EGFR c.2369C>T p.(Thr790Met) in non-small cell lung cancer tissue samples
Source: BMC Cancer. 2020 May 1;20:366. doi: 10.1186/s12885-020-06831-3 (PMC7193365; doi:10.1186/s12885-020-06831-3)
Supplement: Supplementary file 2 — Additional file 2 Supplemental Table S2: Overview of applied test methods and reasons reported for the technical failures observed in the EQA schemes. [file 12885_2020_6831_MOESM2_ESM.pdf]

Supplemental Table 2: Overview of applied test methods and reasons reported for the technical failures observed in the EQA schemes.

Title	Sensitive detection methods are key to identify secondary EGFR c.2369C>T p.(Thr790Met) in non-small cell lung cancer tissue samples.
Journal	BMC Cancer
Authors	Cleo Keppens, Elisabeth MC Dequeker, Etienne Rouleau, Nils ‘t Hart, Lukas Bubendorf, Kelly Dufraing, Céline Garrec, Paul Guéguen, Aude Lamy, Antonio Marchetti, Patrick Pauwels, Ales Ryska, Véronique Tack, Luigi Tornillo, Kaat Van Casteren, Jan H von der Thüsen, Karen Zwaenepoel, Birgit Lissenberg-Witte, Erik Thunnissen, Ed Schuuring
Correspondence	Prof. dr. Ed Schuuring<br>Department of Pathology (HPC EA10)<br>University Medical Center Groningen<br>PO Box 30001, 9700 RB<br>Groningen, the Netherlands<br>Tel: (+31) 50 361 9623<br>Email: e.schuuring@umcg.nl

Supplemental table 2: Overview of applied test methods and reasons reported for the technical failures observed in the EQA schemes.

Scheme year	Sample type	c.2369C>T p.(Thr790Met) variant allele frequency (%)	Method used	Laboratory	Reason for technical failure
**2013**	Cell line	25	Cobas EGFR Mutation detection Test v1 (Roche)	1	Inconclusive result
				2	No amplification
			MAD-EGFR mut (Master Diagnostica S. L.)	3	DNA concentration too low
			EGFR StripAssay (ViennaLab)	4	DNA concentration too low
			EGFR 29 Mutations Detection Kit (AmoyDx)	5	DNA quality too low
			EGFR Mutation Analysis Reagents (Applied Biosystems)	6	DNA concentration too low
			Therascreen EGFR RGQ PCR Kit (Qiagen DxS)	7	Insufficient tissue material for analysis
			Massarray 4 analyser (Sequenom)	8	DNA concentration too low
			MassArray OncoCarta panel (Sequenom)	9	Sample did not pass QC
			Dideoxy sequencing	10	DNA concentration too low
				11	Inconclusive result
				12	No amplification
			Pyrosequencing	13	DNA concentration too low
			High resolution melting	14	No amplification
			Missing data	15	DNA concentration too low
**2014**	Cell line	17	Therascreen EGFR pyro Kit (Qiagen DxS)	16	Inconclusive result
**2015**	Resection	15	Therascreen EGFR RGQ PCR Kit (Qiagen DxS)	17	DNA concentration too low
				18	DNA concentration too low
			Ion AmpliSeq Colon and Lung Cancer Panel (Life technologies)	19	DNA concentration too low
			Pyrosequencing	20	DNA concentration too low
			High resolution melting	21	DNA quality too low
**2016**	Cell line	22	Oncomine Solid Tumour DNA kit (Life Technologies)	16	No amplification
**2017**	Cell line	20	Cobas EGFR Mutation detection Test v2 (Roche)	22	Insufficient neoplastic cells
				23	Insufficient neoplastic cells
			Cobas EGFR Mutation detection Test v1 (Roche)	24	Insufficient neoplastic cells
			Dideoxy sequencing	25	Insufficient neoplastic cells
