# Supplementary material for: Sensitive detection methods are key to identify secondary EGFR c.2369C>T p.(Thr790Met) in non-small cell lung cancer tissue samples
Source: BMC Cancer. 2020 May 1;20:366. doi: 10.1186/s12885-020-06831-3 (PMC7193365; doi:10.1186/s12885-020-06831-3)
Supplement: Supplementary file 3 — Additional file 3 Supplemental Table S3: Performance of different technique types related to time, number of EQA participations, sample type and variant allele frequency. [file 12885_2020_6831_MOESM3_ESM.pdf]

Supplemental Table 3: Performance of different technique types related to time, number of EQA participations, sample type and variant allele frequency.

Title	Sensitive detection methods are key to identify secondary EGFR c.2369C>T p.(Thr790Met) in non-small cell lung cancer tissue samples.
Journal	BMC Cancer
Authors	Cleo Keppens, Elisabeth MC Dequeker, Etienne Rouleau, Nils ‘t Hart, Lukas Bubendorf, Kelly Dufraing, Céline Garrec, Paul Guéguen, Aude Lamy, Antonio Marchetti, Patrick Pauwels, Ales Ryska, Véronique Tack, Luigi Tornillo, Kaat Van Casteren, Jan H von der Thüsen, Karen Zwaenepoel, Birgit Lissenberg-Witte, Erik Thunnissen, Ed Schuuring
Correspondence	Prof. dr. Ed Schuuring<br>Department of Pathology (HPC EA10)<br>University Medical Center Groningen<br>PO Box 30001, 9700 RB<br>Groningen, the Netherlands<br>Tel: (+31) 50 361 9623<br>Email: e.schuuring@umcg.nl

Supplemental table 3: Performance of different technique types related to time, number of EQA participations, sample type and variant allele frequency.

	Commercial kit (n=656)					Next-generation sequencing (n=239)†					Non-commercial method (n=293)‡					Total (n=1188)§				
	# tests	% test with correct outcome	% tests with false-negative result	% tests with wrong mutation	% tests with a technical failure	# tests	% test with correct outcome	% tests with false-negative result	% tests with wrong mutation	% tests with a technical failure	# tests	% test with correct outcome	% tests with false-negative result	% tests with wrong mutation	% tests with a technical failure	# tests	% test with correct outcome	% tests with false-negative result	% tests with wrong mutation	% tests with a technical failure
EQA scheme year																				
2013	56	77.6	22.4	0.0	12.5^{***}	5	66.7	33.3	0.0	40.0^{**}	44	61.5^{**}	38.5^{**}	0.0	11.4^{**}	105	70.3^{***}	29.7^{***}	0.0	13.3^{***}
2014	291	78.6^{***}	20.3^{***}	1.0	0.3^{**}	23	95.7	4.3	0.0	0.0	161	83.2	16.1	0.6	0.0^{**}	475	81.0^{***}	18.1^{***}	0.8	0.2^{***}
2015	58	89.3	10.7	0.0	3.4	34	87.9^{**}	9.1^{*}	3.0	2.9	22	65.0	35.0	0.0	9.1	114	84.4	14.7	0.9	4.4
2016	34	100.0^{**}	0.0^{**}	0.0	0.0	37	100.0	0.0	0.0	2.7	12	91.7	8.3	0.0	0.0	83	98.8^{***}	1.2^{***}	0.0	1.2
2017 (Jun.)	61	73.8^{**}	26.2^{**}	0.0	0.0	30	96.7	3.3	0.0	0.0	16	81.3	18.8	0.0	0.0	107	81.3	18.7	0.0	0.0
2017 (Oct.)	52	100.0^{**}	0.0^{**}	0.0	5.8	32	100.0	0.0	0.0	0.0	18	94.1	5.9	0.0	5.6	102	99.0^{***}	1.0^{***}	0.0	3.9
2018	104	100.0^{***}	0.0^{***}	0.0	0.0	78	100.0	0.0	0.0	0.0	20	100.0^{*}	0.0^{*}	0.0	0.0	202	100.0^{***}	0.0^{***}	0.0	0.0^{*}
Number of EQA participations‖																				
1st	309	79.3^{***}	19.7^{***}	1.0	2.9	43	90.0^{*}	10.0^{**}	0.0	7.0^{*}	141	71.1^{***}	28.1^{***}	0.7	4.3	493	77.9^{***}	21.2^{***}	0.8	3.8^{**}
2nd	181	88.3	11.7	0.0	0.6	59	96.6	1.7	1.7	1.7	99	86.9	13.1	0.0	0.0	339	89.3	10.4	0.3	0.6^{*}
3rd	70	89.9	10.1	0.0	1.4	33	100.0	0.0	0.0	0.0	22	95.2	4.8	0.0	4.5	125	93.5^{*}	6.5^{*}	0.0	1.6
4th	42	90.0	10.0	0.0	4.8	40	97.5	2.5	0.0	0.0	11	90.0	10.0	0.0	9.1	93	93.3	6.7	0.0	3.2
5th	30	96.7	3.3	0.0	0.0	30	100.0	0.0	0.0	0.0	14	100.0	0.0	0.0	0.0	74	98.6^{***}	1.4^{**}	0.0	0.0
6th	20	100.0	0.0	0.0	0.0	24	100.0	0.0	0.0	0.0	6	100.0	0.0	0.0	0.0	50	100.0^{**}	0.0^{**}	0.0	0.0
7th	4	100.0	0.0	0.0	0.0	10	100.0	0.0	0.0	0.0	/	/	/	/	/	14	100.0	0.0	0.0	0.0
Sample type																				
Cell line	312	91.7^{***}	7.6^{***}	0.7	3.5^{**}	77	98.6	1.4	0.0	3.9	189	86.3^{**}	13.1^{**}	0.5	3.2	578	90.9^{***}	8.6^{***}	0.5	3.5^{**}
Resection	344	79.5	20.2	0.3	0.6	162	96.3	3.1	0.6	0.6	104	71.6	28.4	0.0	1.9	610	82.6	17.0	0.3	0.8
Variant allele frequency																				
11%-20%	211	88.8	11.2	0.0	2.8	111	95.5	3.6	0.9	0.9	87	83.3	15.5	1.2	3.4	409	89.5^{*}	10.0^{*}	0.5	2.4
21%-30%	300	77.8^{***}	21.5^{***}	0.7	2.3	82	97.5	2.5	0.0	3.7	152	75.5^{*}	24.5^{*}	0.0	3.3	534	80.2^{***}	19.5^{***}	0.4	2.8
>40%	145	95.2^{***}	4.1^{***}	0.7	0.0	46	100.0	0.0	0.0	0.0	54	92.6^{*}	7.4^{*}	0.0	0.0	245	95.5^{***}	4.1^{***}	0.4	0.0^{**}

Technical failures are represented with respect to the total number of tests. Correct results, false-negatives and wrong mutations are calculated in relation to the total number of analyzable tests (total tests minus technical failures). †The category 'next-generation sequencing' includes both commercial and in-house panels. ‡Non-commercial methods include in-house sequencing methods that are non-NGS based. §Two tests were excluded as no method information was available, bringing the total analyzed tests on 1188. The two excluded tests were performed in 2013 during a 1st EQA participation, on cell line material with an allele frequency of 25%, and resulted in one correct result and one technical failure. ‖The first and second distribution round of the Lung 2017 scheme are counted as two separate participations (4 months apart). Abbreviations: #, number; /, not applicable; EQA, external quality assessment; NGS, next-generation sequencing. Chi-squared tests or Fisher's Exact tests (for cell counts below 5) were used to assess significance. *p<0.05, **p<0.01, ***p<0.001.
