# Supplementary material for: Sensitive detection methods are key to identify secondary EGFR c.2369C>T p.(Thr790Met) in non-small cell lung cancer tissue samples
Source: BMC Cancer. 2020 May 1;20:366. doi: 10.1186/s12885-020-06831-3 (PMC7193365; doi:10.1186/s12885-020-06831-3)
Supplement: Supplementary file 4 — Additional file 4 Supplemental Table S4: Performance over time for the 11 most frequently used c.2369C > T p.(Thr790Met) detection methods. [file 12885_2020_6831_MOESM4_ESM.pdf]

Supplemental Table 4: Performance over time for the 11 most frequently used c.2369C>T p.(Thr790Met) detection methods..

Title	Sensitive detection methods are key to identify secondary EGFR c.2369C>T p.(Thr790Met) in non-small cell lung cancer tissue samples.
Journal	BMC Cancer
Authors	Cleo Keppens, Elisabeth MC Dequeker, Etienne Rouleau, Nils ‘t Hart, Lukas Bubendorf, Kelly Dufraing, Céline Garrec, Paul Guéguen, Aude Lamy, Antonio Marchetti, Patrick Pauwels, Ales Ryska, Véronique Tack, Luigi Tornillo, Kaat Van Casteren, Jan H von der Thüsen, Karen Zwaenepoel, Birgit Lissenberg-Witte, Erik Thunnissen, Ed Schuuring
Correspondence	Prof. dr. Ed Schuuring<br>Department of Pathology (HPC EA10)<br>University Medical Center Groningen<br>PO Box 30001, 9700 RB<br>Groningen, the Netherlands<br>Tel: (+31) 50 361 9623<br>Email: e.schuuring@umcg.nl

Supplemental table 4: Performance over time for the 11 most frequently used c.2369C>T p.(Thr790Met) detection methods.

EQA scheme year	Method for c.2369C>T p.(Thr790Met) detection	Commercial kit					Next-generation sequencing			Non-commercial sequencing		
		Therascreen EGFR RGQ PCR Kit (Qiagen)	Cobas EGFR Mutation detection Test v1 (Roche)	Cobas EGFR Mutation detection Test v2 (Roche)	Therascreen EGFR Pyro Kit (Qiagen)	EGFR Mutation Analysis Kit (EntroGen)	Ion Ampliseq Custom panel - regions selected by the laboratory (Life technologies)	Ion AmpliSeq Colon and Lung Cancer Panel (Life technologies)	TruSight Tumor OncoPanel / TruSight Tumor 15 (Illumina)	Dideoxy sequencing	High-resolution melting	TaqMan-based sequencing (ARMS/LNA/PNA/CAST-PCRs)
2013	# tests performed by method	18	19	/	7	3	/	/	/	32	4	4
2014		79	78	/	50	38	6	/	/	66	38	20
2015		13	17	3	5	8	8	8	/	12	6	1
2016		5	5	10	5	2	6	4	2	1	3	3
2017		13	3	42	13	8	9	9	14	17	7	4
2018		4	2	46	10	4	6	12	16	10	6	/
2013	% test with correct outcome	70.6	94.1	/	85.7	66.7	/	/	/	58.6	100.0	50.0
2014		92.4	65.4	/	85.7	73.7	100.0	/	/	77.3	92.1	70.0
2015		100.0	100.0	100.0	80.0	87.5	100.0	71.4	/	41.7	100.0	100.0
2016		100.0	100.0	100.0	100.0	100.0	100.0	100.0	100.0	100.0	66.7	100.0
2017		53.8	100.0	97.5	53.8	75.0	100.0	100.0	92.9	87.5	100.0	100.0
2018		100.0	100.0	100.0	100.0	100.0	100.0	100.0	100.0	100.0	100.0	/
2013	% tests with false-negative result	29.4	5.9	/	14.3	33.3	/	/	/	41.4	0.0	50.0
2014		3.8	34.6	/	14.3	26.3	0.0	/	/	22.7	7.9	25.0
2015		0.0	0.0	0.0	20.0	12.5	0.0	28.6	/	58.3	0.0	0.0
2016		0.0	0.0	0.0	0.0	0.0	0.0	0.0	0.0	0.0	33.3	0.0
2017		46.2	0.0	2.5	46.2	25.0	0.0	0.0	7.1	12.5	0.0	0.0
2018		0.0	0.0	0.0	0.0	0.0	0.0	0.0	0.0	0.0	0.0	/
2013	% tests with wrong mutation	0.0	0.0	/	0.0	0.0	/	/	/	0.0	0.0	0.0
2014		3.8	0.0	/	0.0	0.0	0.0	/	/	0.0	0.0	5.0
2015		0.0	0.0	0.0	0.0	0.0	0.0	0.0	/	0.0	0.0	0.0
2016		0.0	0.0	0.0	0.0	0.0	0.0	0.0	0.0	0.0	0.0	0.0
2017		0.0	0.0	0.0	0.0	0.0	0.0	0.0	0.0	0.0	0.0	0.0
2018		0.0	0.0	0.0	0.0	0.0	0.0	0.0	0.0	0.0	0.0	/

Supplemental table 4: Performance over time for the 11 most frequently used c.2369C>T p.(Thr790Met) detection methods. ***(Continued)***

EQA scheme year	Method for c.2369C>T p.(Thr790Met) detection	Commercial kit					Next-generation sequencing			Non-commercial sequencing		
		Therascreen EGFR RGQ PCR Kit (Qiagen)	Cobas EGFR Mutation detection Test v1 (Roche)	Cobas EGFR Mutation detection Test v2 (Roche)	Therascreen EGFR Pyro Kit (Qiagen)	EGFR Mutation Analysis Kit (EntroGen)	Ion Ampliseq Custom panel - regions selected by the laboratory (Life technologies)	Ion AmpliSeq Colon and Lung Cancer Panel (Life technologies)	TruSight Tumor OncoPanel / TruSight Tumor 15 (Illumina)	Dideoxy sequencing	High-resolution melting	TaqMan-based sequencing (ARMS/LNA/PNA/CAST-PCRs)
2013	% tests with a technical failure	5.6	10.5	/	0.0	0.0	/	/	/	9.4	25.0	0.0
2014		0.0	0.0	/	2.0	0.0	0.0	/	/	0.0	0.0	0.0
2015		15.4	0.0	0.0	0.0	0.0	0.0	12.5	/	0.0	16.7	0.0
2016		0.0	0.0	0.0	0.0	0.0	0.0	0.0	0.0	0.0	0.0	0.0
2017		0.0	33.3	4.8	0.0	0.0	0.0	0.0	0.0	5.9	0.0	0.0
2018		0.0	0.0	0.0	0.0	0.0	0.0	0.0	0.0	0.0	0.0	/

Technical failures are represented with respect to the total number of tests. Correct results, false-negatives and wrong mutations are calculated in relation to the total number of analyzable tests (total tests minus technical failures). The percentage of correct and incorrect results and technical failures across all scheme years and individual methods is given in **Supplemental Table 3**. Because of the low number of tests performed by a specific method in a given year, results are presented on a descriptive level only. Analysis methods are represented as reported by the participants in the electronic datasheets. Abbreviations: #, number; /, not applicable; ARMS, Amplification Refractory Mutation System; CAST, Competitive allele-specific TaqMan; EGFR, epidermal growth factor receptor; EQA, external quality assessment; LNA, locked nucleic acid; PCR, polymerase chain reaction; PNA, peptide nucleic acid.
