# Supplementary material for: Sensitive detection methods are key to identify secondary EGFR c.2369C>T p.(Thr790Met) in non-small cell lung cancer tissue samples
Source: BMC Cancer. 2020 May 1;20:366. doi: 10.1186/s12885-020-06831-3 (PMC7193365; doi:10.1186/s12885-020-06831-3)
Supplement: Supplementary file 5 — Additional file 5 Supplemental Table S5: Use and performance of the different methods for c.2369C > T p.(Thr790Met) detection between 2013 and 2018. [file 12885_2020_6831_MOESM5_ESM.pdf]

Supplemental Table 5: Use and performance of the different methods for c.2369C>T p.(Thr790Met) detection between 2013-2018.

Title	Sensitive detection methods are key to identify secondary EGFR c.2369C>T p.(Thr790Met) in non-small cell lung cancer tissue samples.
Journal	BMC Cancer
Authors	Cleo Keppens, Elisabeth MC Dequeker, Etienne Rouleau, Nils ‘t Hart, Lukas Bubendorf, Kelly Dufraing, Céline Garrec, Paul Guéguen, Aude Lamy, Antonio Marchetti, Patrick Pauwels, Ales Ryska, Véronique Tack, Luigi Tornillo, Kaat Van Casteren, Jan H von der Thüsen, Karen Zwaenepoel, Birgit Lissenberg-Witte, Erik Thunnissen, Ed Schuuring
Correspondence	Prof. dr. Ed Schuuring<br>Department of Pathology (HPC EA10)<br>University Medical Center Groningen<br>PO Box 30001, 9700 RB<br>Groningen, the Netherlands<br>Tel: (+31) 50 361 9623<br>Email: e.schuuring@umcg.nl

Supplemental table 5: Use and performance of the different methods for c.2369C>T p.(Thr790Met) detection between 2013-2018.

Method for c.2369C>T p.(Thr790Met) detection	Scheme years method was used						# tests	% test with correct outcome	% tests with false-negative result	% tests with wrong mutation	% tests with a technical failure
	2013	2014	2015	2016	2017	2018					
Commercial kit	x	x	x	x	x	x	656	85.2	14.3	0.5	2.0
Therascreen EGFR RGQ PCR Kit (Qiagen)*	x	x	x	x	x	x	132	86.8	10.9	2.3	2.3
Cobas EGFR Mutation detection Test v1 (Roche)*	x	x	x	x	x	x	124	76.9	23.1	0.0	2.4
Cobas EGFR Mutation detection Test v2 (Roche)*			x	x	x	x	101	99.0	1.0	0.0	2.0
Therascreen EGFR Pyro Kit (Qiagen)*	x	x	x	x	x	x	90	83.1	16.9	0.0	1.1
EGFR Mutation Analysis Kit (EntroGen)*	x	x	x	x	x	x	63	77.8	22.2	0.0	0.0
Idylla EGFR Mutation Assay (Biocartis)				x	x	x	24	100.0	0.0	0.0	0.0
EGFR StripAssay (ViennaLab)	x	x	x	x			17	56.3	43.8	0.0	5.9
Myriapod Lung Status (Diatech Pharmacogenetics			x		x	x	16	100.0	0.0	0.0	0.0
EGFR 29 Mutations Detection Kit (AmoyDx)	x		x	x	x	x	13	91.7	8.3	0.0	7.7
EGFR TKI response (Diatech Pharmacogenetics)	x	x					13	46.2	53.8	0.0	0.0
Easy EGFR (Diatech Pharmacogenetics)				x	x	x	12	100.0	0.0	0.0	0.0
Myriapod Cancer Status (Diatech Pharmacogenetics)	x	x	x				8	100.0	0.0	0.0	0.0
PNAClamp EGFR Mutation Detection Kit (Panagene)	x	x	x		x		8	62.5	37.5	0.0	0.0
EGFR XL StripAssay (ViennaLab)					x	x	6	100.0	0.0	0.0	0.0
CLART CMA EGFR (Genomica)			x		x	x	5	80.0	20.0	0.0	0.0
EGFR Mutation Analysis Reagents (Applied Biosystems)	x	x					4	100.0	0.0	0.0	25.0
GML Seqfinder EGFR Sequecing Kit		x	x				4	100.0	0.0	0.0	0.0
PentaPanel (Diatech Pharmacogenetics)			x	x	x		4	100.0	0.0	0.0	0.0
Therascreen EGFR29 Mutation Kit (Qiagen DxS)	x	x					4	75.0	25.0	0.0	0.0
Infiniti Plus EGFR Assay (AutoGenomics)		x					3	100.0	0.0	0.0	0.0
Pan cancer panel (Asuragen)						x	2	100.0	0.0	0.0	0.0
Insider EGFR (Evrogen)			x	x			2	100.0	0.0	0.0	0.0

Supplemental table 5: Use and performance of the different methods for c.2369C>T p.(Thr790Met) detection between 2013-2018. ***(Continued)***

Method for c.2369C>T p.(Thr790Met) detection *(continued)*	Scheme years method was used						# tests	% test with correct outcome	% tests with false-negative result	% tests with wrong mutation	% tests with a technical failure
	2013	2014	2015	2016	2017	2018					
MAD-EGFR mut (Master Diagnostica S. L.)	x						1	0.0	0.0	0.0	100.0
Next-generation sequencing	x	x	x	x	x	x	239	97.0	2.6	0.4	1.7
Ion Ampliseq Custom panel - regions selected by the laboratory (Life technologies)*		x	x	x	x	x	35	100.0	0.0	0.0	0.0
Ion AmpliSeq Colon and Lung Cancer Panel (Life technologies)*			x	x	x	x	33	93.8	6.3	0.0	3.0
TruSight Tumor OncoPanel / TruSight Tumor 15 (Illumina)*				x	x	x	32	96.9	3.1	0.0	0.0
Oncomine Solid Tumour DNA kit (Life Technologies)			x	x	x	x	16	100.0	0.0	0.0	6.3
Custom panel (unspecified)	x	x	x	x		x	15	86.7	13.3	0.0	0.0
Actionable Mutations panel (Qiagen)			x		x	x	14	100.0	0.0	0.0	0.0
TruSeq Amplicon Cancer Panel (Illumina)			x		x	x	12	100.0	0.0	0.0	0.0
In-house primers		x	x	x	x	x	12	91.7	0.0	8.3	0.0
Tumor Hotspot MASTR Plus (Multiplicom)			x		x	x	11	100.0	0.0	0.0	0.0
Ion AmpliSeq Colon and Lung Cancer Panel v2 (Life technologies)				x	x		5	100.0	0.0	0.0	0.0
Ion Ampliseq Lung Cancer Panel (Life Technologies)		x		x			5	100.0	0.0	0.0	0.0
Oncomine Focus Assay (Life Technologies)					x	x	5	100.0	0.0	0.0	0.0
SeqCap EZ Choice (KAPA Hyperplus Workflow) (Nimblegen)					x	x	4	100.0	0.0	0.0	0.0
Ion Ampliseq Cancer Hotspot Panel v2 (Life Technologies)			x			x	4	100.0	0.0	0.0	0.0
MassArray OncoCarta panel (Sequenom)	x	x		x			4	100.0	0.0	0.0	25.0
TruSight Tumor 26 (Illumina)				x		x	4	100.0	0.0	0.0	0.0
MassArray custom panel (Sequenom)		x	x				4	100.0	0.0	0.0	0.0
TruSeq Custom Amplicon (Illumina)				x	x		4	100.0	0.0	0.0	0.0
Massarray 4 analyser (Sequenom)	x			x	x		3	100.0	0.0	0.0	33.3
Accel-Amplicon™ Plus EGFR Pathway Panel						x	2	100.0	0.0	0.0	0.0
Comprehensive Thyroid and Lung (CTL) Kit (Archer)						x	2	100.0	0.0	0.0	0.0
GeneRead QIAact Lung DNA UMI Panel (Qiagen)						x	2	100.0	0.0	0.0	0.0

Supplemental table 5: Use and performance of the different methods for c.2369C>T p.(Thr790Met) detection between 2013-2018. ***(Continued)***

Method for c.2369C>T p.(Thr790Met) detection *(continued)*	Scheme years method was used						# tests	% test with correct outcome	% tests with false-negative result	% tests with wrong mutation	% tests with a technical failure
	2013	2014	2015	2016	2017	2018					
SiReTM (Genedin)						x	2	100.0	0.0	0.0	0.0
TruSight Tumor Cancer panel (Illumina)			x				2	100.0	0.0	0.0	0.0
Custom Panel (Qiagen)						x	2	100.0	0.0	0.0	0.0
Access Array System (Fluidigm)				x			1	100.0	0.0	0.0	0.0
Life technologies (Unspecified)	x						1	0.0	100.0	0.0	0.0
Sentosa®SQ NSCLC Panel (Vela Diagnostics)				x			1	100.0	0.0	0.0	0.0
GeneRead DNAseq Custom Panel v2 (Qiagen)				x			1	100.0	0.0	0.0	0.0
Ion Ampliseq Custom panel v2 - regions selected by the laboratory (Life technologies)				x			1	100.0	0.0	0.0	0.0
Non-commercial method	x	x	x	x	x	x	293	81.1	18.6	0.4	2.7
Dideoxy sequencing*	x	x	x	x	x	x	138	73.1	26.9	0.0	2.9
High-resolution melting*	x	x	x	x	x	x	64	93.5	6.5	0.0	3.1
TaqMan-based sequencing (ARMS/LNA/PNA/CAST-PCRs)*	x	x	x	x	x		32	75.0	21.9	3.1	0.0
Pyrosequencing	x	x	x	x	x		19	82.4	17.6	0.0	10.5
Sanger sequencing		x		x	x	x	16	100.0	0.0	0.0	0.0
Fragment analysis	x	x				x	15	80.0	20.0	0.0	0.0
SNaPshot	x	x		x			5	100.0	0.0	0.0	0.0
RT-PCR		x	x				4	100.0	0.0	0.0	0.0
Missing data	x						2	100.0	0.0	0.0	50.0
Grand Total	x	x	x	x	x	x	1190	86.6	13.0	0.4	2.2

Technical failures are represented with respect to the total number of tests. Correct results, false-negatives and wrong mutations are calculated in relation to the total number of analyzable tests (total tests minus technical failures). A combination of all scheme years (2013-2018) is represented. Missing data: no method information was available. *A breakdown of the performance during each scheme year for the most widely used methods per technique type is given in **Supplemental Table 4**. Abbreviations: #: number.
