# Supplementary material for: Sensitive detection methods are key to identify secondary EGFR c.2369C>T p.(Thr790Met) in non-small cell lung cancer tissue samples
Source: BMC Cancer. 2020 May 1;20:366. doi: 10.1186/s12885-020-06831-3 (PMC7193365; doi:10.1186/s12885-020-06831-3)
Supplement: Supplementary file 6 — Additional file 6 Supplemental Figure S1: Ranked biserial correlation between variant allele frequencies and incorrect outcomes (panel A) or technical failures (panel B). [file 12885_2020_6831_MOESM6_ESM.pdf]

Supplemental Figure 1: Ranked biserial correlation between variant allele frequencies and incorrect outcomes (panel A) or technical failures (panel B).

Title	Sensitive detection methods are key to identify secondary EGFR c.2369C>T p.(Thr790Met) in non-small cell lung cancer tissue samples.
Journal	BMC Cancer
Authors	Cleo Keppens, Elisabeth MC Dequeker, Etienne Rouleau, Nils 't Hart, Lukas Bubendorf, Kelly Dufraing, Céline Garrec, Paul Guéguen, Aude Lamy, Antonio Marchetti, Patrick Pauwels, Ales Ryska, Véronique Tack, Luigi Tornillo, Kaat Van Casteren, Jan H von der Thüsen, Karen Zwaenepoel, Birgit Lissenberg-Witte, Erik Thunnissen, Ed Schuuring
Correspondence	Prof. dr. Ed Schuuring<br>Department of Pathology (HPC EA10)<br>University Medical Center Groningen<br>PO Box 30001, 9700 RB<br>Groningen, the Netherlands<br>Tel: (+31) 50 361 9623<br>Email: e.schuuring@umcg.nl

Supplemental figure 1: Ranked biserial correlation between variant allele frequencies and incorrect outcomes (panel A) or technical failures (panel B).

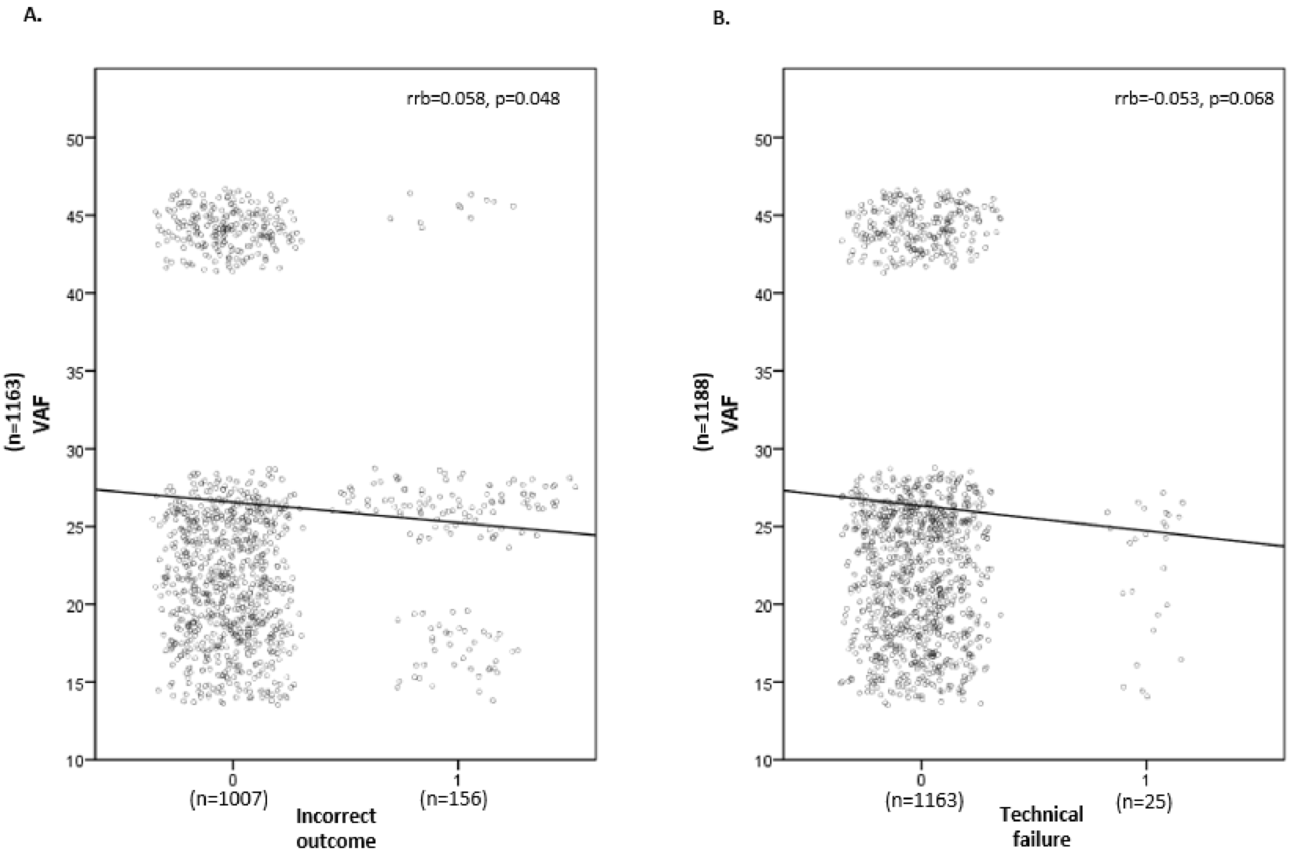

Statistics were performed using IBM SPSS Statistics version 25 (IBM, Armonk, NY, USA). Jitter was applied to minimize overlapping data points. A ranked biserial correlation was performed with the occurrence of an error (false-negative or wrong mutation) or technical failure as dichotomous variable (1:present versus 0:not present) and VAF as ordinal variable **A**. For correlation of the VAF with false-negative and incorrect mutations, only analyzable cases were taken into account, i.e. 1188 tests with exclusion of 25 technical failures. **B**. For correlation of the technical failures all tests were taken into account i.e. 1188 tests. Abbreviations: rrb, ranked biserial correlation coefficient; VAF, variant allele frequency.
